# Species and tissue-specificity of prokinetic, laxative and spasmodic effects of *Fumaria parviflora*

**DOI:** 10.1186/1472-6882-12-16

**Published:** 2012-03-10

**Authors:** Najeeb-ur-Rehman, Malik Hassan Mehmood, Adnan J Al-Rehaily, Ramzi AA Mothana, Anwar H Gilani

**Affiliations:** 1 Natural Product Research Division, Department of Biological and Biomedical Sciences, The Aga Khan University Medical College, Karachi 74800, Pakistan; 2 Department of Pharmacognosy, College of Pharmacy, King Saud University, Riyadh, Saudi Arabia; 3 Department of Pharmacognosy|, Faculty of Pharmacy, Sana'a-University, Sana'a, Yemen

## Abstract

**Background:**

*Fumaria parviflora *Linn. (*Fumariaceae*), is a small branched annual herb found in many parts of the world including Saudi Arabia and Pakistan. This study was designed to provide pharmacological basis for the medicinal use of *Fumaria parviflora *in gut motility disorders.

**Methods:**

The *in-vivo *prokinetic and laxative assays were conducted in mice. Isolated intestinal preparations (ileum and jejunum) from different animal species (mouse, guinea-pig and rabbit) were separately suspended in tissue baths containing Tyrode's solution bubbled with carbogen and maintained at 37°C. The spasmogenic responses were recorded using isotonic transducers coupled with PowerLab data acquisition system.

**Results:**

The aqueous-methanol extract of *Fumaria parviflora *(Fp.Cr), which tested positive for the presence of alkaloids, saponins, tannins and anthraquinones showed partially atropine-sensitive prokinetic and laxative activities in the *in-vivo *in mice at 30 and 100 mg/kg. In the *in-vitro *studies, Fp.Cr (0.01-1 mg/ml) caused a concentration-dependent atropine-sensitive stimulatory effect both in mouse tissues (jejunum and ileum), and rabbit jejunum but had no effect in rabbit ileum. In guinea-pig tissues (ileum and jejunum), the crude extract showed a concentration-dependent stimulatory effect with higher efficacy in ileum and the effect was partially blocked by atropine, indicating the involvement of more than one types of gut-stimulant components (atropine-sensitive and insensitive). This could be a plausible reason for the greater efficacy of Fp.Cr in gut preparations of guinea-pig than in rabbit or mouse.

**Conclusions:**

This study shows the prokinetic, laxative and spasmodic effects of the plant extract partially mediated through cholinergic pathways with species and tissue-selectivity, and provides a sound rationale for the medicinal use of *Fumaria parviflora *in gut motility disorders such as, indigestion and constipation. This study also suggests using different species to know better picture of pharmacological profile of the test material.

## Background

In traditional medicine, gut motility disorders such as, indigestion and constipation are considered root cause of ill health [1], where self-medication using natural products is common amongst the public [2]. It is not uncommon that constipation usually accompanies with indigestion and different classes of chemical drugs, such as prokinetics and laxatives are used in the conventional medicine for these disorders [3]. On the other hand, herbal remedies (known to contain multiple chemicals) are considered relatively safe and useful in such disorders, thus a single plant offers multiple therapeutic benefits [4].

*Fumaria parviflora *Linn. (*Fumariaceae*), locally known in Saudi Arabia as 'Homaira', is a small branched annual herb found in many parts of the world including Middle East [5] and South Asia [6]. In the Greco-Arab (Unani) traditional medicine, the plant is widely used in gut disorders such as, indigestion, constipation, abdominal cramps and diarrhea [5,6]. However, the plant has not been studied for its medicinal use in indigestion or constipation except a preliminary *in-vitro *study showing a cholinergic activity on the part of another species (*Fumaria indica*) of this genus [7]. In this study, we showed for the first time that *Fumaria parviflora *possesses prokinetic and laxative properties in the *in-vivo *models and the detailed study on the possible mode of action was carried out using the *in-vitro *experiments involving different species and tissues.

Phytochemical studies on *Fumaria parviflora *have shown the presence of alkaloids, such as, adlumidiceine, coptisine, fumariline, parfumine, protopine [8], fumaranine, fumaritine, paprafumicin, paprarine [9], fumarophycine, cryptopine, sanactine, stylopine, bicuculline, adlumine, perfumidine and dihydrosanguirine [10]. However to the best of our knowledge, none of the reported compounds or the parent plant has been studied for its laxative or prokinetic activity.

## Methods

### Plant material

The aerial parts of *Fumaria parviflora *were collected from Al-Souda area in Abha, Saudi Arabia in March, 2010. The plant was authenticated by Dr. Mohammad Yusuf, Taxonomist at the College of Pharmacy, King Saud University and the specimen has been preserved at the herbarium of the College of Pharmacy (voucher # 15515), King Saud University, Riyadh, Saudi Arabia, and also at the Natural Product Research Division, Department of Biological and Biomedical Sciences, The Aga Khan University, Karachi with voucher # Fp-AP-23-10-99.

### Extraction procedure

By following a previously described method [11], the aerial parts of *Fumaria parviflora *were soaked in the aqueous-methanol (30:70) for 3 days and filtered through muslin cloth and Whatman (Maidstone, UK) No.1 filter paper simultaneously. This procedure was repeated three times, and all the filtrates were pooled and evaporated in rotary evaporator (model RE-111, Buchi, Flawil, Switzerland) under reduced pressure to finally obtain the crude extract. The yield of thick dark brown past-like mass was 26.6% w/w.

### Phytochemical screening

Phytochemical investigation of the crude extract of Fp.Cr was carried out qualitatively for the presence of alkaloids, saponins, tannins and anthraquinones as plant constituents according to a standard method [12].

### Chemicals

Acetylcholine perchlorate (ACh), atropine sulphate, carbachol (CCh), histamine hydrochloride, 5-hydroxytryptamine (5-HT), pyrilamine maleate and hexamethonium chloride were purchased from Sigma-Aldrich Chemicals Company (St Louis, MO, USA). SB203186 (1-piperidinylethyl-1H-indole-3-carboxylate) was purchased from Tocris (Ballwin, MO, USA). All chemicals used were of the analytical grade available and solubilized in distilled water.

### Animals

BALB/c mice (weighing 20-25 g), guinea-pigs (weighing 400-600 g) and local breed rabbits (weighing 1-1.5 kg) of either sex, were housed at the animal house of the Aga Khan University under a controlled environment (23-25°C). The animals were kept in respective standard cages and were fasted accordingly before starting the experiments, while in routine they had free access to feed and water. The experiments were performed with the rulings of the Institute of Laboratory Animal Resources, Commission on Life Sciences, National Research Council [13] and approved by the Ethical Committee of the Aga Khan University.

### *In-vivo *experiments

#### Charcoal meal gut transit test

Mice fasted for 12 h were divided into different groups, each containing six animals. One group was treated with saline (10 ml/kg), which served as a negative control and the next group was administered (CCh, 1 mg/kg) as the positive control. The next two groups were treated with increasing doses of Fp.Cr (30 and 100 mg/kg, orally, p.o.), acting as the test groups. After 15 min of treatment, each animal received 0.3 ml of charcoal meal in the form of suspension in distilled water containing 10% gum acacia, 10% vegetable charcoal and 20% starch. The animals were sacrificed following 30 min of treatment, and the abdomen immediately cut opened to excise the whole small intestine. The length of the small intestine and the distance between the pylorus region and the front of the charcoal meal was measured to obtain the charcoal transport ratio or percentage. In order to assess the involvement of ACh-like prokinetic effect of the extract and CCh, respectively, further groups of mice were pretreated with atropine (10 mg/kg, intraperitoneal, i.p.) 15 min prior the administration of the extract or CCh [14].

### Laxative activity test

Mice fasted for 6 h before the experiment were placed individually in cages lined with clean filter paper. The animals were divided into seven groups, each containing six animals; the first group acted as the negative control and was administered saline (10 ml/kg, p.o.), while the next group received CCh (1 mg/kg, i.p.), which served as the positive control. The next two third and fourth groups received oral doses of Fp.Cr (30 and 100 mg/kg, respectively). To explore the possible mechanism of the laxative effect of the extract, three separate groups of mice were pretreated with atropine (10 mg/kg, i.p.) 1 h before administration of the extract or CCh. After 18 h, the feces production (total number of feces and total number of wet feces per group) in all animals was counted, and the percentage increase in wet feces relative to that of total fecal output was considered as the laxative effect [15].

### *In-vitro *experiments

Gut preparations from mouse, guinea-pig and rabbit were obtained after sacrificing the animals through cervical dislocation; the abdomen was cut open, required tissues were identified and isolated out [1,14,16], and the required tissue preparations of 2-3 cm length were mounted in 10 ml tissue baths containing the Tyrode's solution, maintained at 37 °C and aerated with a mixture of 5% carbon dioxide and 95% oxygen (carbogen). The composition of Tyrode's solution (mM) was KCl 2.68, NaCl 136.9, MgCl_2 _1.05, NaHCO_3 _11.90, NaH_2_PO_4 _0.42, CaCl_2 _1.8, and glucose 5.55 (pH 7.4). A preload of 1 g was applied to each tissue, and the contractile responses were recorded using isotonic transducer 50-6360 (Harvard Apparatus, Holliston, MA, USA) coupled with either a student oscillograph (Harvard Apparatus) or PowerLab (ML-845) data acquisition system (AD Instruments; Sydney, Australia) and a computer using chart software (version 5.3). The tissues were allowed to equilibrate for a period of 30 min, and then stabilized with a sub-maximal concentration of ACh (0.3 μM). The tissues were presumed stable only after the reproducibility of the said responses. The plant extract was examined later for any spasmodic activity on the ileum and jejunum preparations of mouse, guinea-pig and rabbit at concentrations ranging from 0.01 to 5.0 mg/ml.

### Statistical analysis

All the data except EC_50 _values are expressed as mean ± standard error of mean (S.E.M.), while "n" represents number of animals/experiments). The median effective concentrations (EC_50 _values) are geometric means with 95% confidence intervals (CIs). One way analysis of variance (ANOVA) followed by Dunnett's test or unpaired *t-*test was used to assess the laxative activity, while one-way ANOVA followed by Tukey's test was employed for the effect of plant extract in charchoal meal transit. The concentration-response curves (CRCs) to the agonist responses were analysed by non-linear regression and two-way ANOVA followed by Bonferroni's post-test correction or unpaired *t*-test was used for multiple comparisons of CRCs with the respective control. All the graphing, calculations and statistical analysis were performed using GraphPad Prism 4 for windows (GraphPad Software, San Diego, California, USA).

## Results

### Phytochemical analysis

Preliminary phytochemical analysis of Fp.Cr revealed the presence of alkaloids, saponins, anthraquinones and tannins.

### *In-vivo *findings

#### Effect of Fp.Cr on charcoal meal GI transit in mice

**Figure 1:**
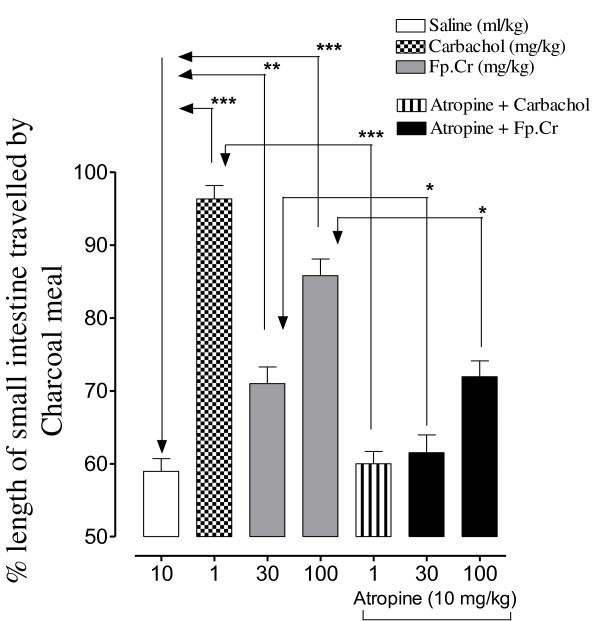
**Bar diagram showing the dose-dependent effect of the crude extract of *Fumaria parviflora *(Fp.Cr) on travel of charcoal meal through small intestine of mice, in the absence and presence of atropine**. *p < 0.05, **p < 0.01 and ***p < 0.001, one-way ANOVA followed by Tukey's test.

Fp.Cr, dose-dependently (30-100 mg/kg) propelled the charcoal meal through the small intestine of mice (Figure 1), thus showing prokinetic effect. The distance travelled by the vehicle control in saline was 58.9 ± 1.7% (mean ± S.E.M, n = 6) of total length of small intestine, while the positive control group receiving CCh (1 mg/kg) significantly enhanced the movement (p < 0.001 versus saline) of charcoal meal to 96.3 ± 1.8%. The plant extract at the doses of 30 and 100 mg/kg significantly (p < 0.001) enhanced the movement of charcoal meal to 71 ± 2.2% and 85.8 ± 2.3% respectively, when compared with saline treated group (58.9 ± 1.7%). Pretreatment of animals with atropine (1 mg/kg) significantly reduced the prokinetic effect of the plant extract and CCh as shown in Figure 1.

### Laxative activity

The treatment with Fp.Cr (30 and 100 mg/kg) produced 61.2 ± 2.2% and 71 ± 3.2% (mean ± S.E.M, n = 6) wet feces in mice. The positive control, CCh (1 mg/kg) produced 79.5 ± 3.3% wet feces, while the saline treated group did not form any wet feces. Pretreatment of animals with Fp.Cr (30 and 100 mg/kg) reduced wet feces to 30 ± 4.3% and 10.6 ± 5.0% respectively, while the reduction in CCh-induced wet feces (6.0 ± 3.8%) was more pronounced as evident in Table 1.

**Table 1:** Laxative effect of the crude extract of *Fumaria parviflora *(Fp.Cr) without and with atropine in mice

**Group No**.	Treatment	Dose (mg/kg)	Mean defecation/group	Mean number of wet feces/group	Mean % of wet feces
1	Saline (p.o., ml/kg)	10	3 ± 0.36	0	0

2	Carbachol (i.p.)	1	11.5 ± 0.5***	9.1 ± 0.5	79.5 ± 3.3

3	Fp.Cr (p.o.)	30	8.3 ± 0.9**	5.2 ± 0.6	61.2 ± 2.2
		
4		100	11.1 ± 0.3***	8.0 ± 1.0	71.0 ± 3.2

5	Carbachol (i.p.) + Atropine (i.p.)	1 + 10	4.0 ± 0.5***	0.3 ± 0.2	6.0 ± 3.8

6	Fp.Cr (p.o.) + Atropine (i.p.)	30 + 10	5.3 ± 0.9**	1.5 ± 0.2	30.0 ± 4.3
		
7		100 + 10	4.6 ± 0.6***	0.5 ± 0.2	10.6 ± 5.0

Values shown are mean ± S.E.M, n = 6. **p < 0.01 and ***p < 0.001 show a comparison of group # 2,3 and 4 vs. group # 1 (One-way ANOVA followed by Dunnett's test), group # 5 vs. group # 2, group # 6 vs. group # 3 and group # 7 vs. group # 4 (unpaired *t-*test). The term p.o represent per oral, while i.p is for intraperitoneal injection.

### *In-vitro *findings

#### Effect of Fp.Cr on ileum preparation of different animals

**Figure 2:**
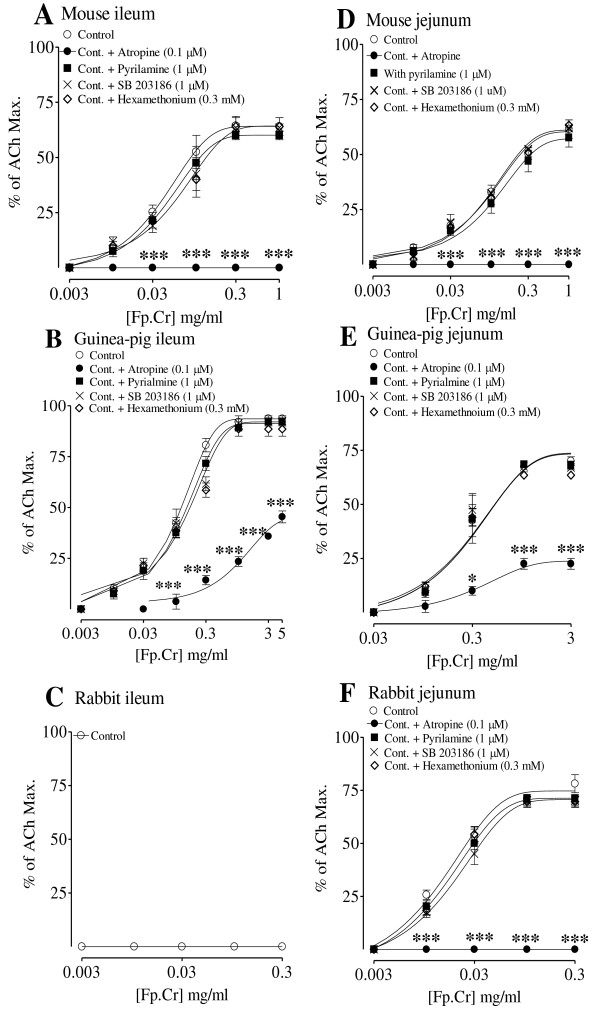
**The stimulatory effects of the crude extract of *Fumaria parviflora *(Fp.Cr) without and with atropine (0.1 μM), pyrilamine (1 μM), hexamethonium (0.3 mM) and SB203186 (1 μM) in isolated ileum and jejunum preparations of (A, D) Mouse, (B, E) Guinea-pig and (C, F) Rabbit**. The values shown are mean ± S.E.M of 4-7 experiments. *p < 0.05 and ***p < 0.001 (Two-way ANOVA, followed by bonferoni post-test correction or unpaired *t*-test).

Fp.Cr at 0.01-0.3 mg/ml caused a stimulatory effect in isolated mouse ileum with efficacy of 62.5 ± 2.2% of ACh maximum, as shown in Figure 2A. Pretreatment of the tissue with atropine (0.1 μM) abolished the spasmogenic effect of Fp.Cr, while pretreatment of the tissue with hexamethonium, pyrilamine, or SB203186 had no effect (Figure 2A). The values determined in Figure 2A have been acquired from 4-7 separate experiments carried out on the isolated tissues of 4 animals.

In guinea-pig ileum, the plant extract caused stimulatory effect in a concentration-dependent (0.01-3 mg/ml) manner with maximum effect (93.2 ± 1%) reaching close to that of ACh maximum. Pretreatment of the tissue with atropine (0.1 μM), but not with hexamethonium, pyrilamine, or SB203186, partially blocked (p < 0.001) the spasmogenic effect of Fp.Cr (Figure 2B).

In rabbit ileum, Fp.Cr did not show any stimulatory effect on tested concentrations of 0.003-0.3 mg/ml (Figure 2C).

### Effect of Fp.Cr on jejunum preparation of different animals

In mouse jejunum, Fp.Cr, caused a concentration-dependent (0.01-1 mg/ml) stimulant effect with efficacy of 62 ± 3% being less potent (p < 0.05) than in mouse ileum. Pretreatment of the tissue with atropine (0.1 μM) blocked the spasmogenic effect of Fp.Cr (Figure 2D).

In guinea-pig jejunum, Fp.Cr (0.1-3 mg/ml) exhibited a spasmodic effect reaching its maximum to 70 ± 0.8% of ACh maximum, which was less than that obtained in ileum (p < 0.05). Like in ileum, pretreatment of tissue with atropine partially blocked the stimulatory effect of Fp.Cr, while pretreatment with hexamethonium, pyrilamine or SB203186 had no influence on the stimulatory effect of Fp.Cr (Figure 2E).

When tested in spontaneously contracting rabbit jejunum, the plant extract exhibited a concentration-dependent (0.01-0.3 mg/ml) stimulatory effect. The efficacy of the spasmogenic effect was 78.1 ± 3% of ACh maximum observed at 0.3 mg/ml. Pretreatment of the tissue with atropine (0.1 μM) completely blocked the spasmogenic effect of Fp.Cr, while the presence of hexamethonium, pyrilamine or SB203186 did not alter its effect (Figure 2F).

## Discussion

When studied for its prokinetic and laxative effects in mice, the plant extract caused propulsion of charcoal meal through small intestine and increased the production of wet feces, similar to the effect of CCh, a standard cholinergic agonist and accelerator of intestinal contents [17]. These gut stimulatory actions of the extract were partially suppressed when studied in the presence of atropine, a muscarinic receptor blocker [18], indicating the presence of some other gut stimulant constituent(s), in addition to ACh-like component(s). ACh is a neurotransmitter of the parasympathetic nervous system and is known to cause gastrointestinal stimulation through the activation of muscarinic receptors [17]; hence, the presence of ACh-like constituents explains its medicinal use in indigestion and constipation.

To further study the possible mode of the observed prokinetic and laxative actions of the extract, the *in-vitro *experiments were conducted. We used two different preparations (jejunum and ileum) from three different species (mouse, rabbit and guinea-pig), based on the previous observations that the plant extract do exhibit tissue and/or species-specific gut stimulatory effect [14,19]. Though efficacy for spasmodic effect of the plant extract varied in intestinal preparations from mouse (jejunum and ileum) and rabbit (jejunum), but its stimulant effect was completely blocked by atropine, thus, showing a common mechanism of gut stimulatory effect through cholinergic action, whereas, the insensitivity of rabbit ileum to stimulatory components of the extract might be due to its tissue selective effects or the presence of some other constituent of opposite mechanism more likely to be expressed in rabbit ileum. Similar tissue specific behavior was also seen in other medicinal herbs like, ginger [20], ispaghula [1] and black pepper [21].

While atropine completely blocked the stimulatory effect of plant extract in the isolated gut tissues of mice, however, only partially blocked the prokinetic and laxative effects of the plant extract in the *in-vivo *studies, which needs explanation. It is possible that the plant extract causes release of some endogenous gut stimulant mediator(s), other than cholinergic in nature.

In guinea-pig tissues, the observed stimulant effect of the plant extract was partially blocked by atropine, indicating that guinea-pig tissues behave differently from those of mouse and rabbit in the nature of gut stimulant effect, and clearly suggests some additional mechanism(s), independent of histamine, nicotine or 5-Hydroxytryptamine (5-HT, serotonin) receptors activation, which was evident by its insensitivity to pyrilamine, a histaminic type-1 (H_1_) receptor blocker [22], hexamethonium, a ganglion blocker [23] or SB203186, a serotonergic receptor antagonist [24]. The other mechanisms known for their gut stimulant property, which have not been ruled out include, platelet activating factor [25], nitric-oxide-donating or releasing compounds [26] and dopaminergic antagonists [27].

Collectively, the data on jejunum and ileum preparations of mouse, rabbit and guinea-pig indicate a species and tissue-selective gut stimulatory effect of Fp.Cr. The observed stimulatory effect of the extract was fully atropine-sensitive in rabbit jejunum and mouse preparations, while guinea-pig tissues showed partial sensitivity to atropine. Rabbit ileum was insensitive to the stimulatory effect of the plant extract, while guinea-pig tissues showed the highest efficacy, ileum being the most sensitive with efficacy close to that of ACh. Such types of species and/or tissue-selective effects of plant extracts have also been reported in earlier studies [1,14,19,20,28,29]. What is the pharmacological basis for this species and/or tissue specific behavior of some plant materials is not clear. Location and distribution of different subtypes of different receptors vary in different species and even difference exists within the tissues from the same species [30]. Furthermore, sometimes, compounds like atropine, which is not selective for any muscarinic receptor subtype, exhibits selective behavior in some species. For example, the potency of atropine is found to be less in rabbit tissues than in other species, which is believed to be due to the presence of atropinase enzyme in rabbit resulting in rapid metabolism of atropine, thus rendering it less active [31]. Similarly, the relatively high efficacy of the plant extract in guinea-pig tissues is possibly because of additional mechanism (non-cholinergic), which may involve activation of receptors, which are not present in other two species studied, though other possibilities cannot be ruled out.

Based on these observations, a suggestion can be made using different gut tissues from more than one species to know the broader picture. It may be worth mentioning that gut stimulant effect of Fp.Cr was significantly (p < 0.001) higher in rabbit jejunum and guinea-pig ileum, when compared with that of *Fumaria indica*, another species of *Fumaria *genus, which showed atropine-sensitive stimulant effects with no species or tissue selective behavior [7]. The observed variation in stimulatory effects of Fp.Cr (collected from Saudi Arabia), and *Fumaria indica *(collected from Pakistan), in guinea-pig ileum and rabbit jejunum could be either due to different plant-species or because of the effect of regional and environmental factors. The chemical composition and biological activity profile in the same plant grown in different regions are known to differ [32,33].

The plant has been shown to possess anthelmintic activity [34] and co-existence of laxative effect might be of added value in the expulsion of helminths, as the helminthes are known to promote developing gut disorders such as, abdominal pain, diarrhea, and constipation [35].

The presence of alkaloids, saponins [36] and anthraquinones [3] as the plant constituents, which are known to possess gut stimulatory properties, may explain the gut stimulant actions of the plant extract, though further studies are required to know the specific chemical(s) responsible for the tested biological activities.

## Conclusion

This study showed that *Fumaria parviflora *possesses prokinetic and laxative activities of in mice, which were partially mediated through a cholinergic pathway. In the *in-vitro *studies, the observed spasmodic effect of the plant extract involved at least two pathways (atropine-sensitive and insensitive) with species and tissue-selectivity, guinea-pig tissues being the most sensitive. This study provides sound pharmacological basis to the medicinal use of the plant in gut motility disorders, like indigestion and constipation, and suggests using more than one species to draw a meaningful conclusion, as no single species can truly represent human picture.

## Competing interests

The authors declare that they have no competing interests.

## Authors' contributions

AHG designed the project and supervised the study. NR carried out the experimental work, data analysis, literature search and drafted manuscript. MHM helped in study design, analysis of data and preparing draft manuscript. AJA and RAAM selected, identified and procured the plant material and corrected the manuscript for publication. All authors read and approved the final manuscript for publication.

## Pre-publication history

The pre-publication history for this paper can be accessed here:

http://www.biomedcentral.com/1472-6882/12/16/prepub
